# Entertainments and Receptions

**Published:** 1867-06

**Authors:** 


ENTERTAINMENTS AND RECEPTIONS.

Dr. Murphy also announced, besides the entertainment last evening, the following

